# Psychosocial Attributes of Housing and Their Relationship With Health Among Refugee and Asylum-Seeking Populations in High-Income Countries: Systematic Review

**DOI:** 10.3389/phrs.2023.1605602

**Published:** 2023-05-04

**Authors:** Tessa-Maria Brake, Verena Dudek, Odile Sauzet, Oliver Razum

**Affiliations:** School of Public Health, Bielefeld University, Bielefeld, Germany

**Keywords:** systematic review, social determinants of health, refugees, asylum seekers, housing

## Abstract

**Objectives:** Housing as a social determinant of health should provide not only shelter, but also a feeling of home. We explored psychosocial pathways creating a sense of home and influencing the relationship between housing and health among asylum seekers and refugees (ASR) in high-income countries.

**Methods:** We performed a systematic review. To be included, studies had to be peer-reviewed, published between 1995 and 2022, and focus on housing and health of ASR in high-income countries. We conducted a narrative synthesis.

**Results:** 32 studies met the inclusion criteria. The psychosocial attributes influencing health most often identified were *control*, followed by *expressing status*, *satisfaction*, and *demand*. Most attributes overlap with material/physical attributes and have an impact on ASR’s mental health. They are closely interconnected with each other.

**Conclusion:** Psychosocial attributes of housing play an essential role in the health of ASR; they are closely associated with material/physical attributes. Therefore, future research on housing and health of ASR should routinely study psychosocial attributes, but always in association with physical ones. The connections between these attributes are complex and need to be further explored.

**Systematic Review Registration:** https://www.crd.york.ac.uk/prospero/, identifier CRD42021239495.

## Introduction

The number of forcibly displaced people has been increasing in recent years. At the end of 2021, 89.3 million people were forcibly displaced. This number included 4.6 million asylum seekers and 27.1 million refugees (1). A refugee is defined as someone who is unable or unwilling to return to their country of origin “owing to a well-founded fear of being persecuted for reasons of race, religion, nationality, membership of a particular social group, or political opinion” (*Refugee Convention*, Article 1, Section 2). An asylum seeker is someone who has left their country and is seeking protection from persecution and human rights violations in another country but has not yet been legally recognized as a refugee and is awaiting a decision on their asylum application (2).

In the coming years, further large refugee flows are expected due to extreme weather events caused by climate change (1). With these changes in climatic conditions and demographic patterns, housing is becoming an essential social determinant for the health of the population (3).

Groups, such as ASR, that are racialized or marginalized by, for example, socioeconomic status and/or ethnicity, not only have poorer health, but also experience poorer conditions with respect to social determinants, such as poorer access to adequate housing (4). Addressing housing as a social determinant of health is relevant for improving health and reducing disparities (5). Adequate housing is a crucial indicator for successful integration (6).

### Housing as a Social Determinant of Health

Associations between housing and health can already be found in empirical literature (3, 7, 8). However, the literature indicates that the causal relationships going beyond the direct effects of structural deficiencies of the dwelling are poorly understood (9–11).

According to the WHO (3), healthy housing should provide “*a feeling of home*, *including a sense of belonging*, *security and privacy*.” (3), p. 2. A recent systematic review found significant associations with physical and mental health for a range of context-related housing attributes among ASR, most of which were material/physical in nature (e.g., mold and damp). This relation was intertwined with other attributes, such as discrimination, which is why the authors point to the need for better research tools to examine this more generally (12).

**FIGURE 1:**
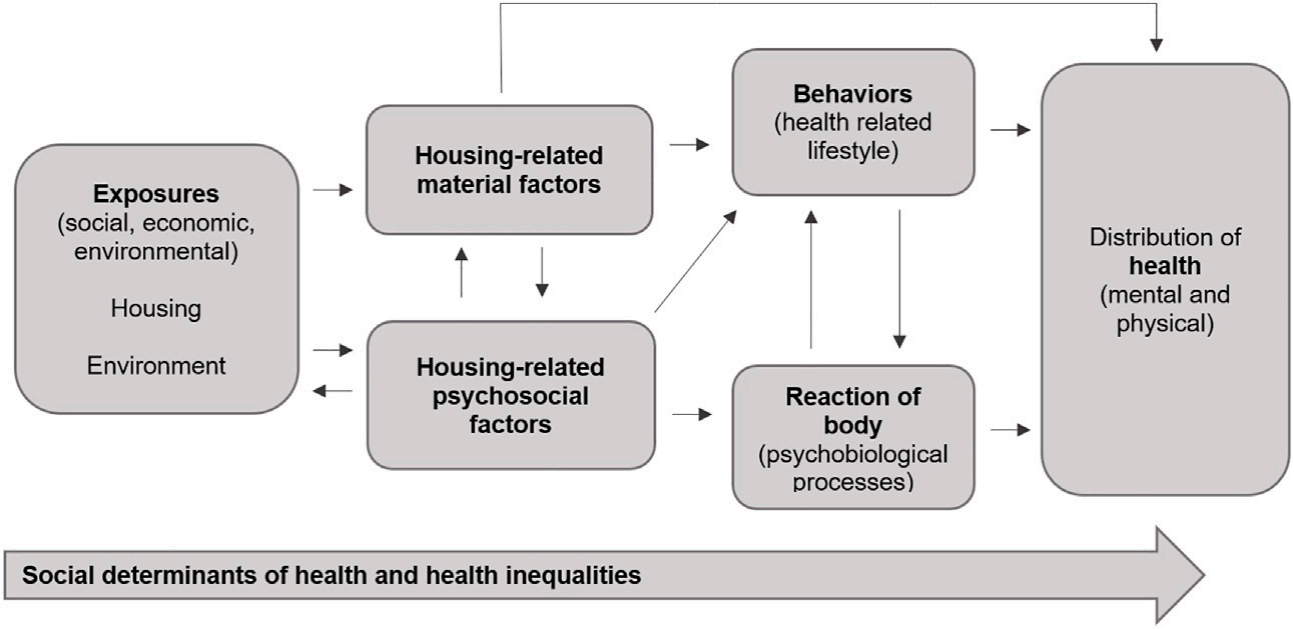
Links between housing and health, including housing-related material/physical and psychosocial factors; own representation following Public Health England, 2017 (Psychosocial Attributes of Housing and Their Relationship With Health Among Refugee and Asylum-Seeking Populations in High-Income Countries: Systematic Review, Bielefeld, Germany. 2022).

The way these attributes are linked is often referred to as *psychosocial pathways*, which are considered important mediators between housing and health (9–11, 13). We consider these attributes, which reflect on the social meanings associated with housing and create a sense of home, as psychosocial attributes in our research. Figure 1 provides an overview. They serve as the starting point for our study. A challenge is to better define the concept of “home” and to make its relevant attributes measurable (14).

### Psychosocial Attributes of Housing

To identify the psychosocial attributes of housing, we refer to a framework by Dunn (15). Dunn collates such attributes in the “meaningful dimension of housing,” one of three key housing dimensions through which social inequalities and health consequences are produced. The dimension is conceptualized around three psychosocial attributes derived from the Job-Demand Control Model (16): personal *control*, *demand*, and *social support*. Considering this, Dunn (15) assumes that these attributes that are most important for health at work will also apply to the place where people spend most of their time: the home. Dunn adds a specific attribute: *satisfaction with housing*. These psychosocial attributes are each defined by a set of indicators (15). *Control* over the dwelling includes indicators of feelings of *comfort and security* (space, privacy, safe neighborhood), while *demand* comprises the *workload* associated with the dwelling (gardening, housework, costs). *Social meanings* cover indicators such as the level of *pride and self-reflection* and the *sense of belonging* to the neighborhood; in this regard, Dunn speaks of “expression of status” (15), p.35 (Supplementary File S1).

Dunn (15) did not focus on particular population subgroups in this work. We argue that these attributes derived from the field of workplace organization also apply to the living situation of ASR. They often have little control over their housing situation due to asylum laws, for example, in Germany, where they are obliged to live in state-mandated initial reception facilities for a given time period (*Asylum Act*, Article 47, Section 1). As this population group already faces several postmigration stressors, we consider it useful to examine the demand associated with the housing environment. We further hypothesize that a lack of social support may negatively affect the health of ASR as they are exposed to social prejudice and loneliness, which in turn affects their integration (17).

We address the following research questions: *1) To what extent are psychosocial attributes of housing taken into account in empirical research assessing the relationship between housing and the health of ASR?* and *2) What is the relationship between psychosocial attributes of housing and the health of ASR?*

## Methods

We conducted a systematic literature review, following the Preferred Reporting Items for Systematic Reviews and Meta-Analysis (PRISMA) checklist (18). The protocol has been registered with PROSPERO (CRD42021239495). Compared to the original protocol, we adjusted the first question for reasons of clarity and extended the search to the year 2022 instead of 2021. We no longer searched the Embase database, but we searched PsycINDEX, SocINDEX and CINAHL.

### Searches

We applied the PECO (Population - Exposure - Comparator - Outcome) framework (19). The population of interest included adult ASR who resettled to a high-income country (HIC). We set no limitation to language, gender, ethnicity, or time since migration. The exposure was related to the housing context and assessed in two stages. First, we considered any material/physical attributes of housing assessed in the studies and their link to health to identify categories (Supplementary File S4). We understood material/physical attributes as attributes that we identify in the literature on the general relationship between housing and health, i.e., all attributes that have not been considered from a psychosocial perspective and their immediate consequences. Second, we assessed which of these attributes falls under psychosocial attributes according to Dunn (15). In order not to exclude potentially relevant studies due to a too narrow concept of health, we included studies assessing any health-relevant outcome referring to physical and mental health, morbidity, and mortality, corresponding to the WHO definition of health (20). We searched PubMed, Web of Science, PsycINFO and PsycINDEX, SocINDEX, CINAHL and the Cochrane Library. We built the search terms (Supplementary File S2) by drawing on the PECO framework. We derived the specific terms from previous housing and/or forced migration research and conducted a pilot search in PubMed.

### Eligibility Criteria

Eligibility criteria are displayed in Table 1. The mixed-methods approach was considered as useful since psychosocial attributes (especially unexpected or previously unknown ones) may be more frequently identified in qualitative research due to the inductive nature of this research method. However, we did not want to overlook quantitative research that may have assessed these attributes, therefore considering both study types eligible for the review. We considered the year 1995 as a time point by which the consequences of the wars that ended in Bosnia and Croatia were increasingly thematized, as preliminary analyses in PubMed showed. Since attributes of housing are measured in different ways in empirical research, we kept eligibility criteria broad and included all studies that may point to any relationship between housing and health in the abstract or title by comprising the term “housing” or synonyms (Supplementary File S2) and “health” or related terms (Supplementary File S2). We excluded studies that focused on homelessness only or defined the housing context only broadly, which would not provide insights into which housing-related attributes are potentially related to health. We did not include studies conducted on internally displaced persons (IDPs) or the general population of (im-)migrants unless the study provided disaggregated data for ASR, as we explicitly wanted to capture experiences with the housing situation of ASR in HICs.

**TABLE 1:** Eligibility criteria (Psychosocial Attributes of Housing and Their Relationship With Health Among Refugee and Asylum-Seeking Populations in High-Income Countries: Systematic Review, Bielefeld, Germany. 2022).

Inclusion criteria	Exclusion criteria
• Peer-reviewed qualitative, quantitative or mixed-methods study published between 1995 and 2022	• Focus of the study is on homelessness only
• Abstract or title includes the term housing or related synonyms (dwelling, shelter, accommodation) and health (or related terms morbidity, mortality, depression, anxiety, post-traumatic stress)	• Housing concept is too broad or unspecified (i.e., if studies did not assess at least one subcomponent of housing)
• Study must be conducted in HIC	• Studies in which target group is comprised of internally displaced persons or general population of (im-)migrants and data for ASR is not disaggregated
• Target population must be ASR (forcibly displaced population)	

### Data Selection and Data Collection Process

Two reviewers (TB, VD) independently screened titles and abstracts of all matches for eligibility. Next, we retrieved texts of potentially eligible studies and again assessed them independently. We discussed inconsistencies during these processes; if necessary, with the help of a third reviewer from the study team.

For data extraction, we used a predesigned form (Supplementary File S3). We piloted it by having both reviewers extract five (approx. 16%) of the studies. After discussing inconsistencies, both reviewers extracted half of the remaining studies.

To identify psychosocial attributes of housing as objectively as possible, we used indicators derived from Dunn (15) (Supplementary File S1). We identified these in three ways: i) direct quantitative elicitation, ii) direct description by study participants or iii) indirect inference from the results. This was the case when study participants referred to attributes without explicitly naming them or when quantitative research measured variables which were similar to the indicators of attributes.

### Quality Assessment and Data Synthesis

We used the Mixed-Methods Appraisal Tool (MMAT) for the assessment of the study quality (21, 22). The MMAT provides different sets of criteria for different study designs: qualitative, quantitative, and mixed methods. Two reviewers (TB, VD) independently applied the MMAT on half of the studies after a pilot phase in which both assessed five studies (approx. 16%) and discussed inconsistencies. To ensure high internal validity, low-quality studies were excluded.

We conducted narrative synthesis since we expected a high heterogeneity of results and a diverse range of methods applied. We followed the Guidance on the Conduct of Narrative Synthesis for Systematic Reviews (23). Accordingly, we conducted a thematic analysis (23). As housing was assessed as a two-level exposure, we first looked for the most important material/physical attributes and listed them in a table. We assigned thematically similar attributes to superordinate housing categories and listed the associated health effects (Supplementary File S4). Second, we listed the psychosocial attributes. We categorized the individual aspects by color (Supplementary File S4) to allow for better classification and overview. Using vote-counting, we investigated the frequency and, thus, the relevance of the attributes. To explore relationships within and between studies, we drew a conceptual map depicting relationships between housing-related attributes and health, also including findings of psychosocial attributes (Supplementary File S5). To assess the robustness of the synthesis, we critically reflected on the process: We discussed the limitations of the method, quality and generalizability, and discrepancies identified in the findings and how we handled them (23).

## Results

**FIGURE 2:**
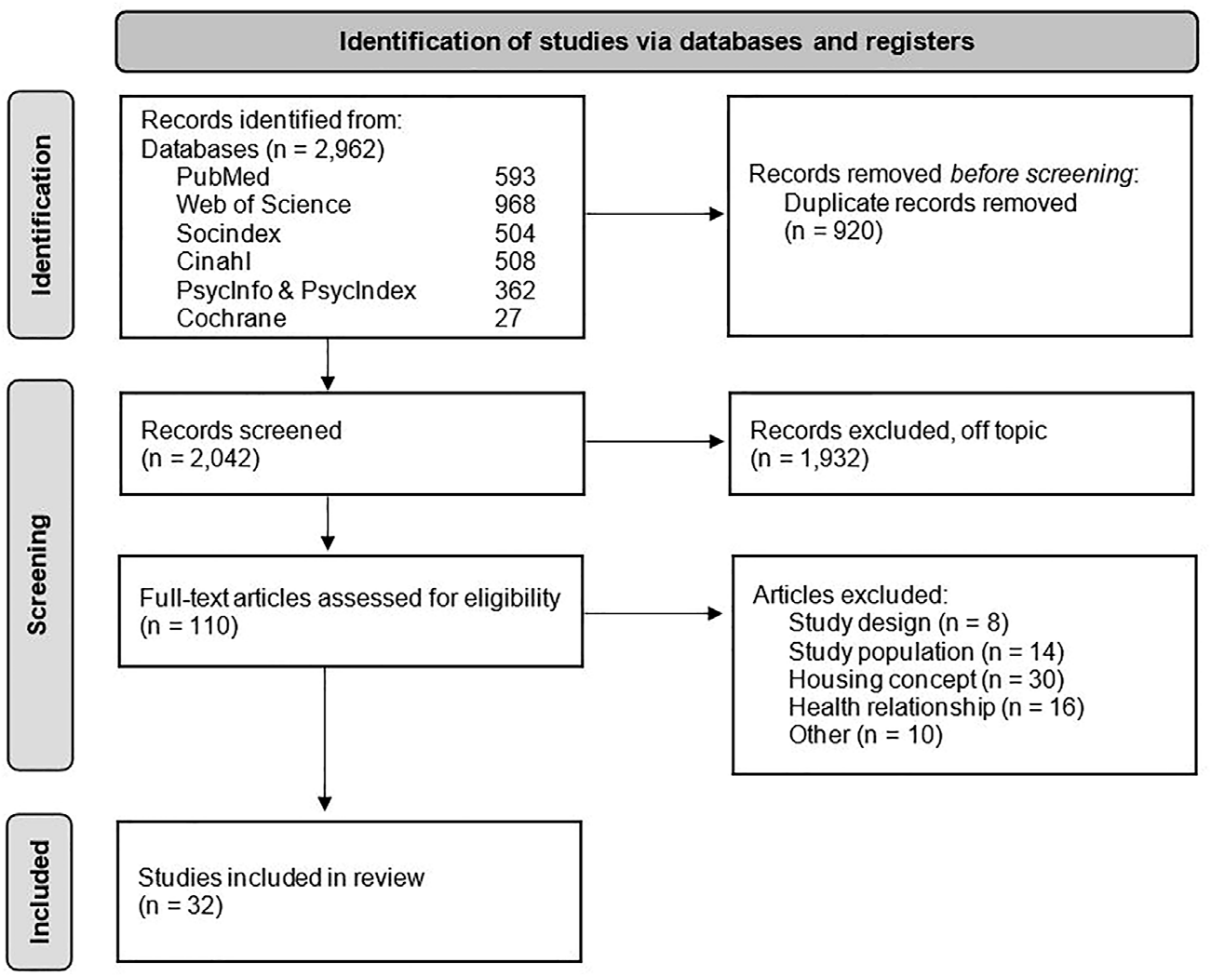
PRISMA Flow chart (Psychosocial Attributes of Housing and Their Relationship With Health Among Refugee and Asylum-Seeking Populations in High-Income Countries: Systematic Review, Bielefeld, Germany. 2022).

We included 32 studies in the review (Supplementary File S6). Figure 2 shows the PRISMA flowchart.

### Overview of Studies

The largest proportion of studies was conducted in the UK (*n* = 8) (24–31), followed by Germany (*n* = 6) (32–37) and Australia (*n* = 5) (38–42). One study each was conducted in Canada (43), the Netherlands (44), Austria (45), Ireland (46), Switzerland (47), Norway (48), and Sweden (49). Most studies (*n* = 25) included adult ASR (24–34, 36–38, 40–43, 46, 47, 49–53), and were published between 2015 and 2022 (*n* = 24) (24, 26, 27, 30, 32–38, 40–43, 45–51, 53, 54). The number of participants in qualitative studies ranged from 6 to 106, and in quantitative studies, from 105 to 5,678 persons. Nineteen studies focused on the outcome of mental health (25, 26, 28, 30, 31, 36–38, 41, 43–46, 48–53). We found housing as a main topic in seven studies (25, 31, 33, 40, 42, 44, 50); in the remaining 25 it was a side topic. The most common countries of origin of the study participants were Syria, Afghanistan, Iraq, Iran, Somalia, and Pakistan.

After quality assessment (Supplementary File S7), we classified 27 studies (84.4%) (25–38, 40–47, 49–52, 55) as high and five (15.6%) (24, 39, 48, 53, 54) as moderate regarding their quality. In the subset of quantitative-descriptive studies, the nonresponse rate, reasons and statistical compensation for nonresponse were not stated in ten (25, 26, 29, 32, 38, 39, 43, 44, 53, 54) of 16 studies. The target population and sample were not adequately described in two studies (39, 53). In one study (54), we awarded zero points for the sampling strategy. Three (4, 53, 54) out of the 16 quantitative-descriptive studies were of moderate quality, the others of high quality (25, 26, 32, 33, 35–38, 43, 44, 49, 50, 55). One quantitative non-randomized study (24) was rated as of moderate quality because it did not provide appropriate outcome measures and data. The evaluation of the qualitative studies showed different limitations. The criterion most frequently violated was the link to the research question, which was missing (28, 42, 46, 48). On the other hand, all qualitative studies were of high quality, except one of moderate quality (48).

### Relationship Between Housing and Health From a Psychosocial Perspective

We identified seven categories of material/physical attributes and their immediate consequences: 1) crowding, space, and privacy, 2) housing instability (including security of tenancy, affordability, and residential mobility), 3) safety, 4) physical conditions of/in the dwelling, 5) housing satisfaction, 6) neighborhood and location, and 7) institutional practices in the dwelling or accommodation center (Supplementary File S4).

We found *crowding*, *space*, *and privacy* in 17 (53.1%) (24, 27, 29, 30, 32–35, 37, 40, 42, 44, 45, 47, 51–53) and *housing instability* in 16 (50%) (25, 28–31, 34, 38, 39, 41, 42, 45, 49–53) of the 32 studies. We further identified the category *physical conditions* in nine (28.1%) (27, 29, 34, 40, 42, 45, 47, 54, 55) and *safety* in eight studies (25.0%) (27, 29, 30, 33, 34, 40, 42, 51). We identified fewer housing-related attributes in quantitative studies. Only in terms of *housing satisfaction* quantitative approaches dominated (23.5% quantitative vs. 6.7% qualitative; number referred to: 17 quantitative and 15 qualitative studies in total).

Psychosocial attributes were directly assessed quantitatively in 12.5% (*n* = 4) (26, 29, 36, 43) of the 32 studies, reported by the study participants in eight studies (25%) (27, 30, 31, 34, 40, 42, 46, 52), and indirectly derived from the results by the reviewers in ten studies (31,3%) (24, 29, 30, 33, 41, 45, 47, 48, 50, 51). Psychosocial attributes were present in 20 (62.5%) (24, 26, 27, 29–31, 33, 34, 36, 40–43, 45–48, 50–52) of the 32 studies.

Looking at the thematic analysis (Supplementary File S4), we found some overlap between the material/physical attributes and the psychosocial attributes according to Dunn (15).

We classified the material/physical attributes *lack of privacy and space* as attributes of the category *crowding*, *space*, *and privacy* under the psychosocial attribute *control*.

The material/physical housing-related category *housing instability* included attributes such as *residential mobility* and *housing costs/affordability*. As participants perceived these as burdens, we assigned *affordability* to the psychosocial attribute *demand*, specifically to its indicator *strain of meeting costs*, and *residential mobility* to the psychosocial indicators *worry of frequent move* and *worry of forced move* as part of the psychosocial attribute *control*.

The category *safety*—within the shelter due to (aggressive) strangers and in the neighborhood—was often linked to a fear of crime and victimization. Thus, we assigned it to the corresponding psychosocial indicator *fear of crime/victimization*.

Regarding *neighborhood*, good social relations were also associated with a sense of *belonging* as an indicator of the psychosocial attribute *expressing status*. In one study (40), participants perceived access to green spaces, as an aspect of *location*, as positive for health. We associated this, in turn, with the feeling of wellbeing at home and thus assigned it to the psychosocial indicator *place of refuge*.

The fourth housing-related category, *physical conditions of/in the dwelling*, included attributes such as *gardens* and *outside spaces* which participants saw as a burden. We thus subsumed it under the indicator *housework strain*, as an indicator of the psychosocial attribute *demand*.

In addition, *satisfaction* emerged as a material/physical attribute, which is also defined as a psychosocial attribute according to Dunn (15).

The seventh category was *institutional practices in the dwelling*: when it came to low control regarding *institutional practices*, participants did not feel as comfortable as they would in a “real” home, which we, in turn, attributed to the psychosocial control indicator *place of refuge*. Moreover, participants felt ashamed of these practices and thus their home did not represent who they really were (46). This was consistent with the definition of the psychosocial attribute *expressing status*, specifically its indicators *pride* and *self-reflection*.

The attributes we identified most frequently related to *control*. Of its four indicators, the indicator *place of refuge* was most prevalent (*n* = 13, 40.6%) (24, 27, 30, 31, 33, 34, 40, 42, 46–48, 51, 52), followed by *fear of crime and/or victimization* (*n* = 7, 21.9%) (27, 33, 34, 40, 42, 50, 51), and the indicators *concern about forced* (30, 31, 40, 42) and *frequent moves* (31, 41, 45, 51) (*n* = 4, 12.5%). *Expressing status* was the second most common. While we found its indicators *pride* (46) and *self-reflection* (46) once each (3.1%) in the 32 studies, we identified the *sense of belonging* to the neighborhood six times (18.8%) (29, 33, 40, 42, 48, 50). This was followed by the attribute *demand*. Its indicator *strain of meeting costs* was addressed in 12.5% (*n* = 5) (40, 42, 51, 52) and the indicator *housework strain* in 3.1% (*n* = 1) (33) of the studies. Finally, we identified the indicator *satisfaction* in five studies (15.6%) (26, 29, 33, 36, 43). We identified all indicators except *satisfaction* more frequently in qualitative than in quantitative studies.

### Relationship Between Psychosocial Attributes of Housing and Health

#### Control

We identified the indicator *place of refuge* most commonly regarding health outcomes. A lack of control over the home (24, 33, 34, 42, 46, 48, 53) and/or of a private retreat (24, 27, 30, 34, 40, 42, 47, 51, 52), including a safe place with enough space, negatively affected mental health. Having to share space with others and the resulting lack of privacy led to feelings of anxiety, discomfort, frustration, disempowerment, and sadness (34, 51–53). Participants perceived the lack of control in influencing daily decisions as a psychosocial stressor for mental and physical health (46). Other frequently encountered indicators were *worry/strain of forced* and *frequent moves*. We found problems with forced moves in four studies, all of which related to a lack of control over the home, leading to disempowerment which subsequently affected mental health, or health and wellbeing in general (30, 40, 42, 53). We also identified four studies that pointed to issues related to frequent moves, which were seen as stressors for health (41, 45, 51, 53). The last indicator referring to *control* pointed to *victimization* and *fear of crime*. While four studies had identified fear of crime due to other residents in the accommodation facility (27, 34, 50, 51), five referred to safety aspects in the neighborhood (27, 33, 40, 42, 50). Fear affected mainly mental health, manifesting as anxiety, but also physical health through lack of sleep and stress (27, 33, 40, 42). Fear of violence and witnessing neighborhood and interpersonal violence were also important causes of involuntary moves (50).

#### Expressing Status

The second most important attribute regarding the health of ASR was *expressing status*, especially the indicator *belonging*, addressed in six studies (29, 33, 40, 42, 48, 50). *Feelings of trust* and *confidence* in the neighborhood contributed to feelings of *belonging*, which ASR perceived as positive for their health and wellbeing (40). This also applies to maintaining good neighbor relations, which helped to develop social bridges. In contrast, the experience of discrimination in the neighborhood had negative health consequences and strengthened the intention to move (50). ASR who were less often in contact with locals and neighbors had significantly poorer mental and physical health scores (33), as social isolation led to depression and stress (29). These scores were significantly worse for ASR living in shared accommodation than for those in private accommodation (33).

#### Satisfaction

Associations between satisfaction with the home and the health of ASR were reported in five studies (26, 29, 33, 36, 43). According to a longitudinal study, low satisfaction with housing conditions was significantly associated with moderate and/or severe depressive symptoms (43). In addition, decreased satisfaction with accommodation was significantly associated with higher odds of poor emotional wellbeing (26). Dudek et al. (33) found that ASR living in collective accommodation had the lowest satisfaction with the living situation and thus had significantly poorer mental and physical health scores than ASR living in private accommodations.

#### Demand

We identified four studies which examined the effect of *affordability* on the health of ASR (40, 42, 51, 52). Participants considered the cost of living a fundamental burden causing stress and worry (42) and perceived it as a strain in terms of personal control, hindering them from moving to better/more appropriate housing (40).

## Discussion

We explored the psychosocial attributes of housing among ASR and their role for health.

We identified seven categories of housing-related attributes relevant to health, ranging from *physical conditions, crowding, space, privacy, safety, housing instability, neighborhood/location, institutional practices* up to the *satisfaction* with the home. We could assign most of the material/physical attributes to the categories *crowding/space/privacy* and *housing instability* which may indicate a high importance of these categories for ASR in HICs.

The psychosocial attributes we identified most often referred to *control*, particularly the indicators *place of refuge* and *forced/frequent moves*.

Although the review by Ziersch & Due (12) did not examine the relationship between housing and the health of ASR from a psychosocial perspective, some comparisons can still be drawn. According to the review (12), the main issues of ASR in resettlement countries related to housing were affordability, tenancy insecurity and mobility, discrimination and difficulties in finding accommodation, overcrowding and the quality or condition of housing. We also found problems with overcrowding, insecure tenure and mobility, and affordability. Discrimination and difficulties in finding housing were assigned to several categories in our review, but not listed separately as in their review (12). Housing quality or housing condition can be equated with our category *condition of the dwelling*(s). Furthermore, they found that most studies in resettlement countries focused on mental health and only a small proportion considered housing a main issue (12). This is also reflected in our study. A scoping review on the impact of housing on refugees also highlights that there is a direct link between living in poor and insecure accommodation and poor mental health as well as limited opportunities to build social networks (56).

Regarding the psychosocial perspective, according to Dunn (15), we hypothesized, that the lack of control related to housing opportunities might be associated with adverse health outcomes. Looking at the broader literature, a review (57) found that characteristics of housing quality can influence psychosocial processes, such as lack of control over management practices, which can affect mental health. WHO Europe (58), in its analysis and review of housing and health status in the European Region, showed that perceptions of control over housing conditions were relevant in the prevalence of anxiety or depression. This was mainly related to deprived or inadequate housing conditions (58). These findings of the general population are confirmed by our study on ASR, which found that a lack of control can affect physical and mental health (24, 27, 30, 33, 34, 40, 42, 46, 47, 51–53). Scientific literature shows that this population is mainly affected by poor and inadequate housing conditions (59).

Additionally, we hypothesized that the workload associated with housing might also be perceived as a health-related burden by ASR. However, only one study found that gardening was considered a burden by ASR in private homes (40). This study did not find any association with the health of ASR. The fact that only one study addressed this issue suggests that the strain of housework itself is less relevant for this population. Looking beyond our review, one reason could be that ASR often live in state-provided accommodations where meals and general work around/in the house are prepared or carried out by people working there (60). According to a population-based study in Germany, for example, almost half of the asylum seekers and around 20% of recognized refugees were living in shared accommodations at the end of 2018 (61).

We further considered it reasonable that ASR perceive social stigma regarding their living situation and face challenges building social connections, with negative consequences for their health. According to an empirical study (62), respondents who felt that their home reflected their identity well and were proud of it were more likely to report better general and mental health. In our review, *pride* and *self-reflection* indicators were only identified in one study, suggesting that they are less relevant to the target population. Also, no relationship was highlighted between these aspects and the health of ASR (46). In relation to inviting friends, however, the study by Dudek et al. (33) indicated that living in shared accommodation with little control over the housing situation, including the ability to invite friends, affects both mental and physical health. So, there is a link to the psychosocial attribute *control*. The indicator *belonging* was a crucial psychosocial attribute in our research due to its frequency and relevance to health (12, 29, 33, 40, 50). Looking at the broader literature, social contact between refugees and the local population is a prerequisite for integration; the openness and tolerance of at least a part of the local population can help refugees feel at home and strengthen their intention to stay (63). Additionally, crime and antisocial behavior in housing estates (e.g., drug dealing, gang fights) lead to stress and a lower sense of security and belonging (64).

We also assumed that by analyzing satisfaction levels we can get an idea of the extent to which housing, with the given material structures, has personal meaning for ASR. A German longitudinal panel study found that higher housing satisfaction was associated with better self-rated health (65). Satisfaction with housing was further found to be a strong predictor of wellbeing. In this respect, dissatisfaction with, for example, green spaces or air quality indirectly influenced wellbeing of the participants. Significantly associated with this satisfaction were age, gender, and socio-economic status (58). Our review supports these findings. Hence, decreased satisfaction with current accommodation was significantly associated with higher odds of poor emotional wellbeing (26), and with severe- and/or moderate depression levels (36, 43).

Furthermore, our results show that psychosocial attributes are more associated with mental than physical health. This could be due to the long lag times of many physical (non-communicable) diseases (66).

### Limitations and Strengths

Starting with limitations, we did not conduct a “grey” literature search, screen the references for more potential studies and include other than peer-reviewed articles. This may have incurred publication bias (67) as non-significant results may not have been published (68). If so, we would be overestimating the strength of association between psychosocial attributes and the health of ASR. Because one of the three methods we used to identify psychosocial attributes was to derive them indirectly from study results, we may have been subjective in our assessment which could affect the reproducibility of our approach. This could also have been the case with classifying the material/physical attributes into the seven categories. Contrary to recommendations (21, 69), due to time constraints as one reviewer left the working group, the two reviewers only extracted and assessed five studies independently. The remaining studies were split up, extracted and assessed by one person each. This approach potentially increased the risk of error and the possibility that a single person’s bias influenced data selection. We tried to minimize this problem by frequently communicating about our procedures and discussing them intensively.

We included studies featuring a variety of methods and with heterogeneous results. So, we could only conduct narrative synthesis, including thematic analysis, instead of meta-analysis, which may have reduced transparency and methodological clarity (70, 71). The heterogeneity of studies in terms of design, sample size, methodical approach, and research focus limits their comparability, interpretation, and generalizability.

However, to our knowledge, our review is the first to consider the psychosocial perspective as a pathway between housing and the health of ASR in HICs. By conducting a systematic review, we minimized the risk of *ad hoc* search and bias (72). Adherence to the PRISMA checklist improved the reporting process and ensured a systematic, transparent approach (18). The broad range of databases searched can also be seen as a strength. By not specifying a particular housing concept, we avoided bias that might have resulted from the rejection of potentially relevant studies. For the same reason, we included qualitative studies since we assumed that psychosocial attributes of housing might be likely to emerge from perceptions and experiences. To counteract selection bias, we did not restrict the search in terms of language or full-text availability. Additionally, our study focused only on resettlement in HICs which might reduce the contextual heterogeneity of studies. Finally, a clear definition of psychosocial attributes according to Dunn (15) contributed to a systematic search and strengthens our findings’ reproducibility.

### Conclusion and Recommendations for Action

Our study provides evidence of the influence of psychosocial attributes of housing on ASR health. To conclude, housing in the context of health needs to be considered beyond its purely material/physical structures. Such structures are closely associated with psychosocial attributes which make homes meaningful, in particular, the perceived degree of control over the home.

On this basis, we recommend that housing for ASR offers sufficient space and privacy, as well as opportunities to develop. Affordable housing is an important mental health issue and should hence be given high priority. Mass shelters, in which ASR often spend long periods of time (73), are associated with perceived insecurity and a lack of opportunities for privacy, negatively affecting mental health and wellbeing. Therefore, as a short-term response, opportunities for more privacy must be established until private accommodation becomes available, and social support services need to be strengthened. Furthermore, ASR are often placed in deprived areas, which may be related to increased crime rates in the neighborhood and thus increase the fear of being victimized and having to move. The sense of belonging to the neighborhood is often weak, so its role as an important mediator between housing and health that can promote integration is lost. Accordingly, attention must be paid to neighborhood conditions when allocating housing to ensure healthy, anxiety-free living, and successful integration. As a long-term solution to addressing the broader structural inequalities that constrain the lives of ASR, ASR-led organizations are calling for the abolition/dismantling of mass shelters (74).

Further research should examine the associations between psychosocial attributes and the health of ASR more closely, especially regarding physical health outcomes. The interaction of the attributes with one another should be investigated. In addition, quantitative studies need to cover psychosocial attributes more comprehensively as qualitative findings demonstrate their relevance. However, due to the strong interconnectedness with the material/physical attributes of housing, psychosocial attributes should not be investigated detached from material/physical attributes. Since Dunn’s assumptions are based on the reality of Western societies, particularly Canada, further research is needed specifically on our population of interest, which is diverse and comes from different socio-cultural contexts.

## Abbreviations

ASR, Asylum seekers and refugees; HIC, High-income country; IDP, Internally displaced person.
