# Supplementary material for: Psychosocial Attributes of Housing and Their Relationship With Health Among Refugee and Asylum-Seeking Populations in High-Income Countries: Systematic Review
Source: Public Health Rev. 2023 May 4;44:1605602. doi: 10.3389/phrs.2023.1605602 (PMC10193459; doi:10.3389/phrs.2023.1605602)
Supplement: Supplementary file 1 [file DataSheet1.docx]

**Psychosocial attributes of housing and their relationship with health among refugee and asylum-seeking populations in high-income countries: systematic review**

**Supplementary file 1: Indicators of psychosocial attributes derived from Dunn [14] (own representation) (Bielefeld, Germany, 2022)**

| 1 | **Measures of demand** | |
| --- | --- | --- |
| 1.1 | Housework strain | Degree to which participants perceive any work associated with the home (including gardening) physically or mentally as a strain or burden |
| 1.2 | Strain of meeting costs | Degree to which participants perceive housing costs (including affordability issues) physically or mentally as a strain or burden. (Note: not affordability itself but the individual burden of affordability issues is addressed here) |
|  | | |
| 2 | **Measures of control** | |
| 2.1 | Place of refuge | Degree to which participants feel uncomfortable at home (such as issues of disturbed privacy, lack of retreat or low opportunities to personalize own home) |
| 2.2 | Worry of forced move | Degree to which participants are worried about a forced move (demonstrating a lack of control of stable housing). Note: not the number of forced moves but the feelings about it (such as worry, fear) are addressed here |
| 2.3 | Worry of frequent moves | Degree to which participants perceive high housing mobility (frequent moves) as a burden or strain. Note: not the frequency of moves itself but the perceptions about it are addressed here |
| 2.4 | Fear of crime / victimization | Degree to which participants feel unsecure at home and fear becoming a victim of crime: security / safety issues in the domestic environment (home including neighbourhood) |
|  | | |
| 3 | **Measures of expressing status** | |
| 3.1 | Pride | Degree to which participants are proud to show their homes to visitors |
| 3.2 | Self-reflection | Degree to which participants feel like their homes reflects who they are |
| 3.3 | Belonging | Degree to which participants feel like they belong in their neighbourhood |
|  | | |
| 4 | **General measures** | |
| 4 | Satisfaction | Degree to which participants are satisfied with their housing situation (different components of housing are possible here, as long as it relates to housing / home) |

**Supplementary file 2: Search terms (Bielefeld, Germany, 2022)**

| **PubMed**  (((((((("housing"[Title/Abstract] OR "accommodation"[Title/Abstract] OR "dwelling"[Title/Abstract] OR "shelter"[Title/Abstract] OR "home"[Title/Abstract]) NOT ("ocular"[All Fields] OR "oculars"[All Fields])) NOT ("animals"[MeSH Terms:noexp] OR "animal"[All Fields])) NOT "homeless"[All Fields]) NOT ("homeless persons"[MeSH Terms] OR ("homeless"[All Fields] AND "persons"[All Fields]) OR "homeless persons"[All Fields] OR "homeless"[All Fields] OR "homelessness"[All Fields])) OR ("housing/adverse effects"[MeSH Terms] OR "housing/classification"[MeSH Terms] OR "housing/instrumentation"[MeSH Terms] OR "housing/methods"[MeSH Terms] OR "housing/standards"[MeSH Terms] OR "housing/trends"[MeSH Terms])) NOT "housing, animal"[MeSH Terms]) AND ("humans"[MeSH Terms] AND 1995/01/01:2022/04/20[Date - Publication])  AND  (("health"[Title/Abstract] OR "morbidity"[Title/Abstract] OR "mortality"[Title/Abstract] OR "depression"[Title/Abstract] OR "post-traumatic stress"[Title/Abstract] OR "posttraumatic stress"[Title/Abstract] OR "anxiety"[Title/Abstract]) AND "humans"[MeSH Terms] AND ("humans"[MeSH Terms] AND 1995/01/01: 2022/04/20[Date - Publication]))  AND  (("refugee*"[All Fields] OR ("asylum"[All Fields] OR "asylum s"[All Fields] OR "asylums"[All Fields]) OR "forced migration"[All Fields] OR "forced migrant*"[All Fields] OR "displaced person*"[All Fields] OR "displaced population*"[All Fields] OR "refugees"[MeSH Terms]) AND ("humans"[MeSH Terms] AND 1995/01/01: 2022/04/20[Date - Publication]) AND "humans"[MeSH Terms]) AND "humans"[MeSH Terms]) AND (humans[Filter]) |
| --- |

| **Web of Science**  (TI=hous* OR TI=accommodation* OR TI=dwelling* OR TI=shelter* OR AB=hous* OR AB=accommodation* OR AB=dwelling* OR AB=shelter* NOT ALL=ocular NOT ALL=oculars NOT ALL=homeless NOT ALL=homelessness)  AND  (TI=health OR TI=morbidity OR TI=mortality OR TI=depression OR TI=anxiety OR TI="post traumatic stress" OR AB=health OR AB=morbidity OR AB=mortality OR AB=depression OR AB=anxiety OR AB="post traumatic stress")  AND  (ALL=refugee* OR ALL=asylum OR ALL=("forced migration") OR ALL=("forced migrant*") OR ALL=("displaced person*") OR ALL=("displaced population*")) |
| --- |

| **CINAHL, SOCIndex, PSYCHIndex, PSYCHINfo**  "((TI (hous* OR dwelling* OR shelter* OR accommodation*)) OR (AB (hous* OR dwelling* OR shelter* OR accommodation)) NOT (ocular OR homeless OR homelessness))  AND  ((TI (health OR morbidity OR mortality OR depression OR "post-traumatic stress" OR "posttraumatic stress" OR anxiety)) OR (AB (health OR morbidity OR mortality OR depression OR "post-traumatic stress" OR "posttraumatic stress" OR anxiety)))  AND  ((TX refugee*) OR (TX displaced person*) OR (TX displaced population*) OR (TX forced migration) OR (TX forced migrant*) OR (TX asylum seeker*) OR (TX asylum-seeker*)) Published Date: 19950101-20210420" |
| --- |

| **Cochrane Library**  (housing OR dwelling OR accommodation OR shelter):ti,ab,kw NOT (homeless OR homelessness OR ocular) OR MeSH descriptor: [Housing] explode all trees NOT MeSH descriptor: [Housing, Animal] explode all trees  AND  (health):ti,ab,kw OR (morbidity OR mortality):ti,ab,kw OR ("post-traumatic stress"):ti,ab,kw OR (depression):ti,ab,kw OR (anxiety):ti,ab,kw  AND  (refugee):ti,ab,kw OR ("asylum seeker"):ti,ab,kw OR ("forced migration" OR "forced migrant"):ti,ab,kw OR ("displaced person" OR "displaced population"):ti,ab,kw OR MeSH descriptor: [Refugees] explode all trees |
| --- |

**Supplementary file 3: Predesigned extraction form (excerpt) (Bielefeld, Germany, 2022)**

**
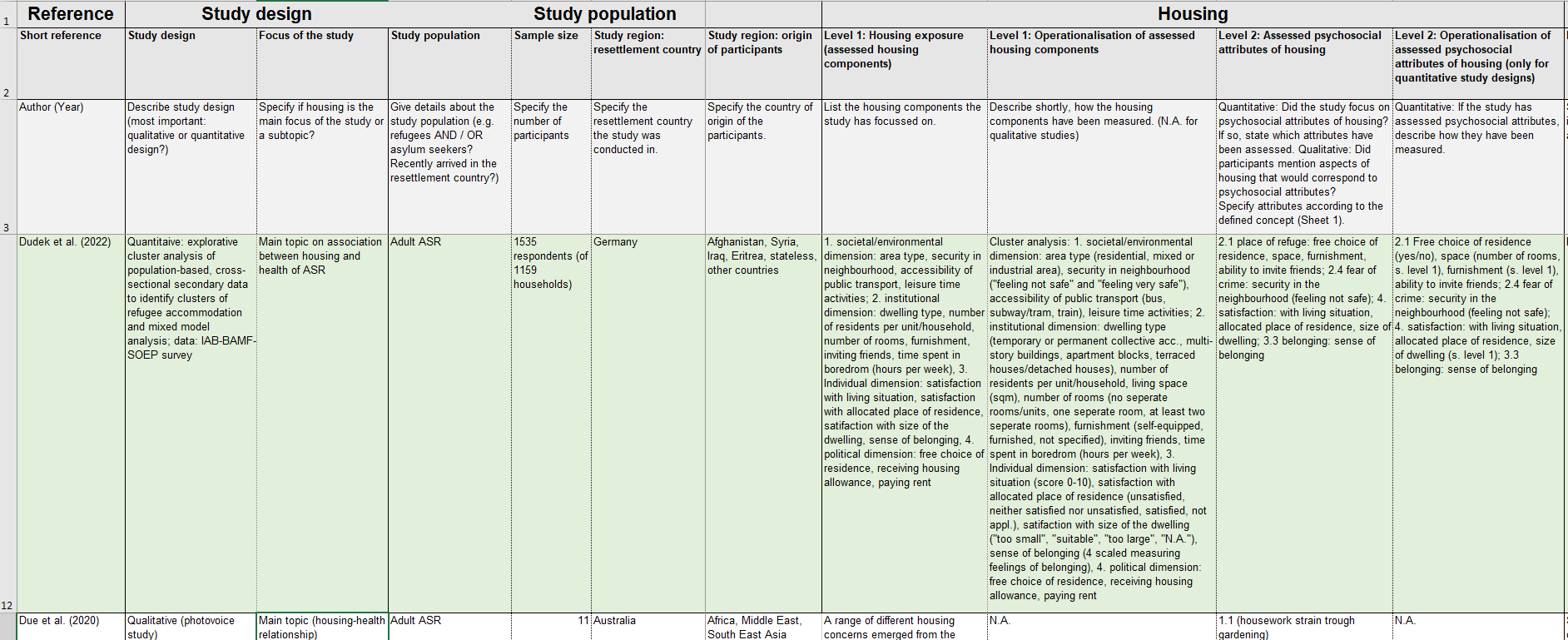
**

**
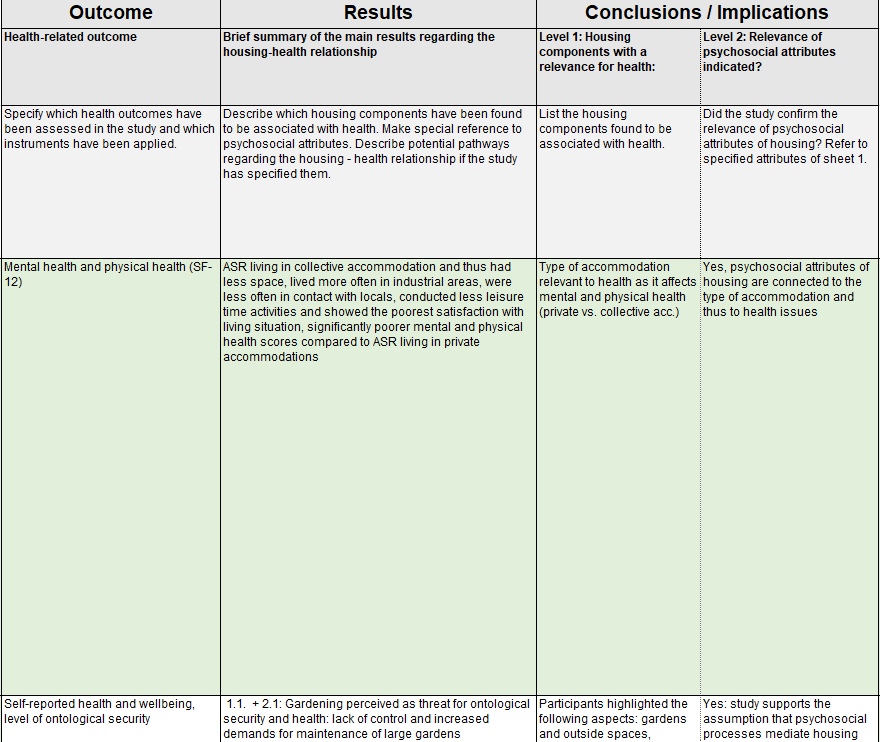
**

**Supplementary file 4: Thematic analysis (Bielefeld, Germany, 2022)**

**
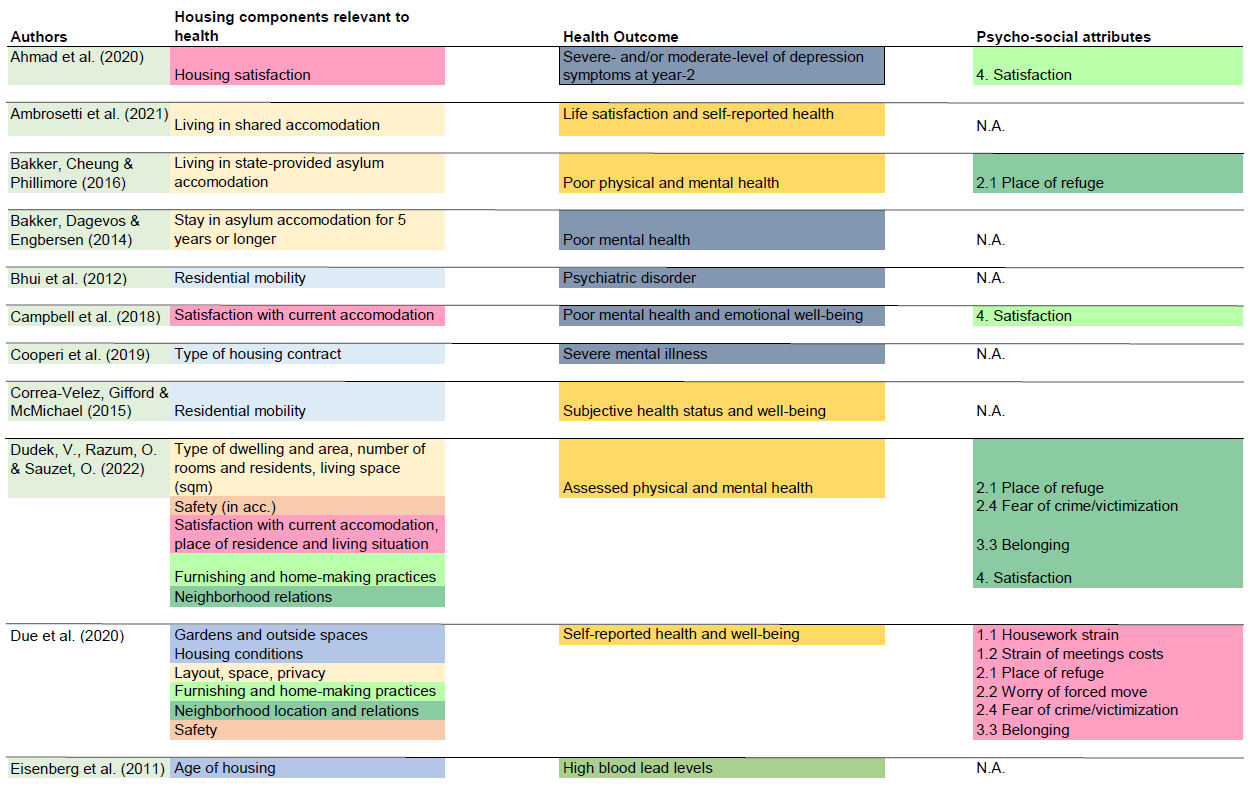
**

**
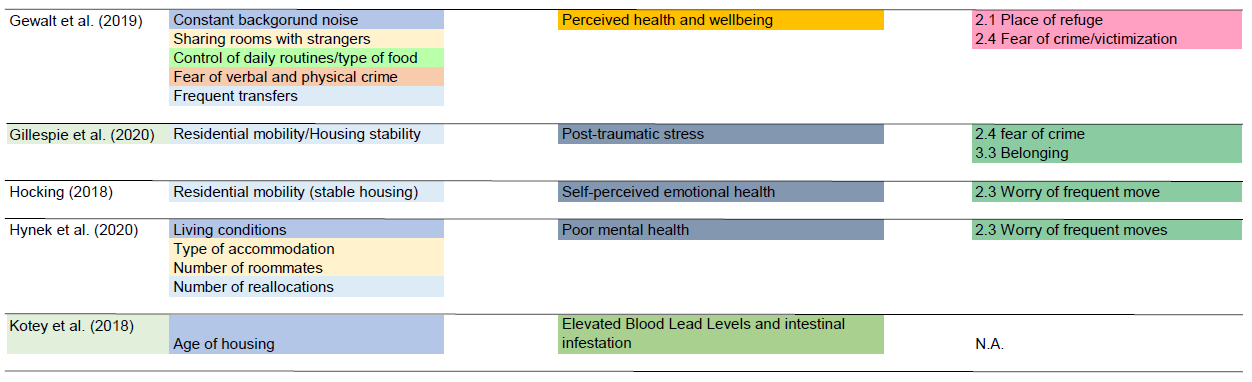
**

**
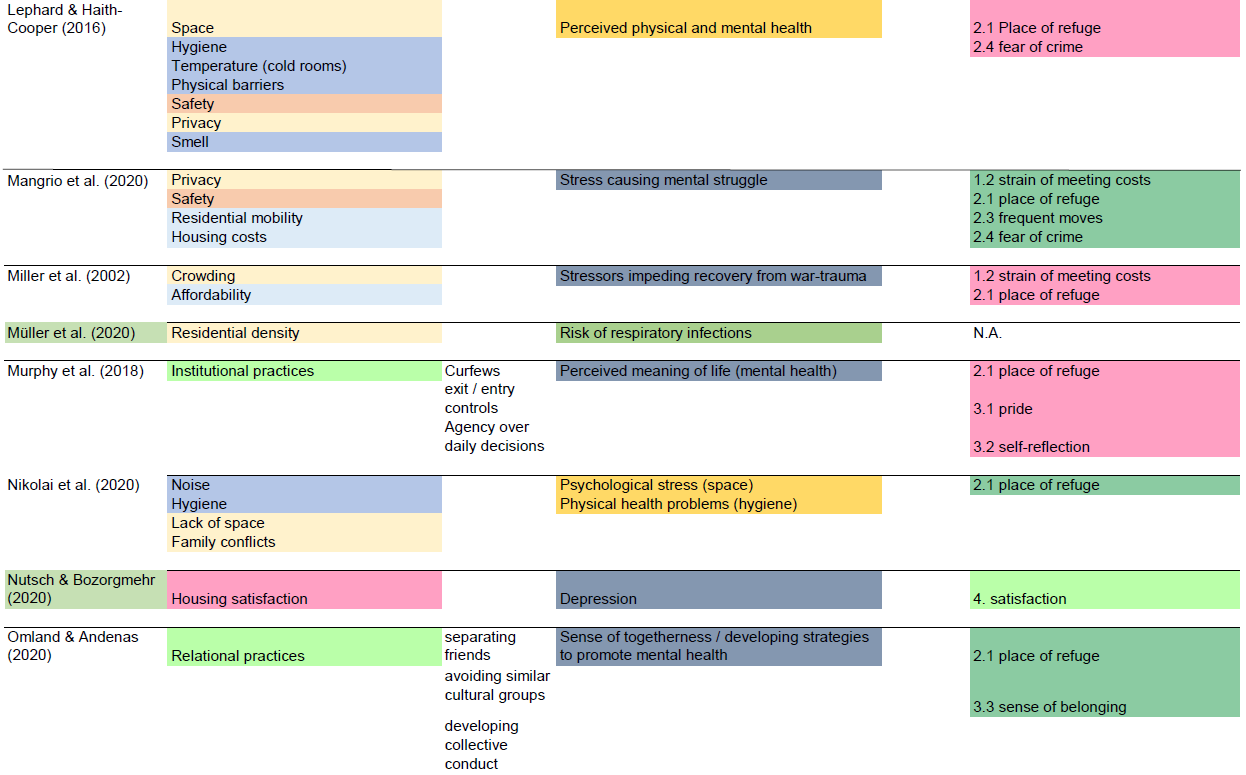
**

**
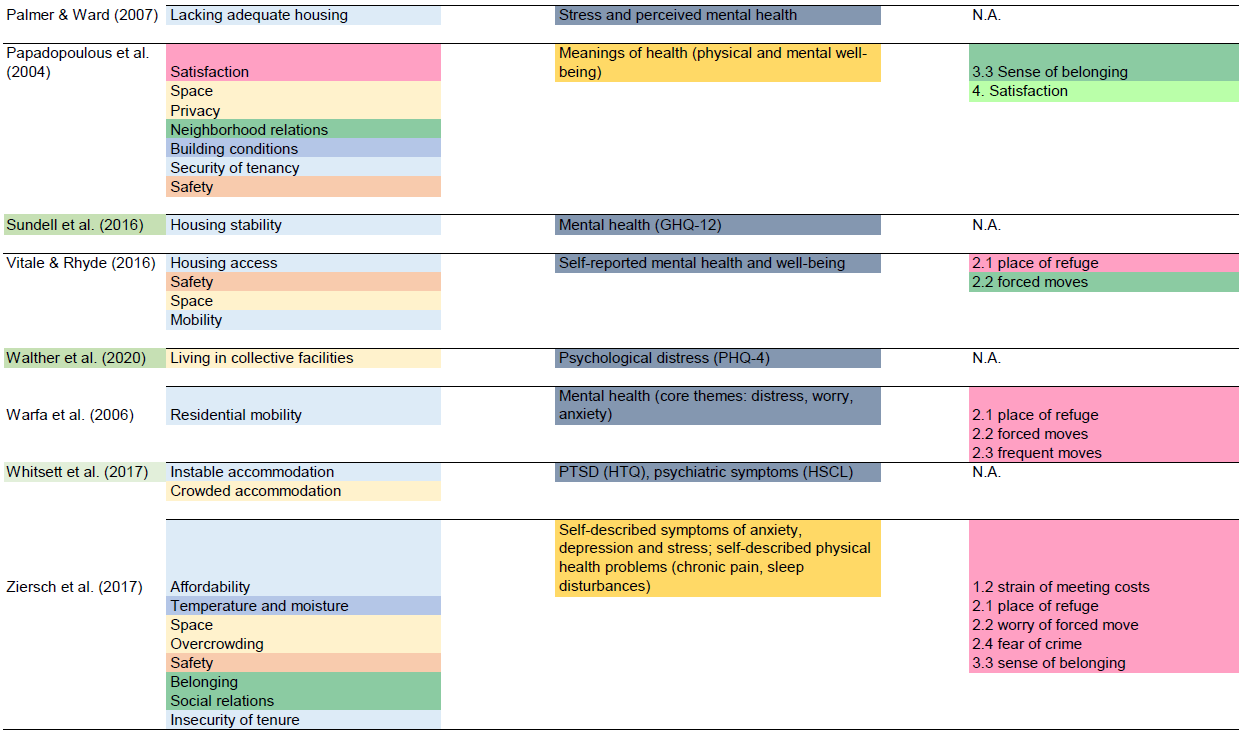
**

**
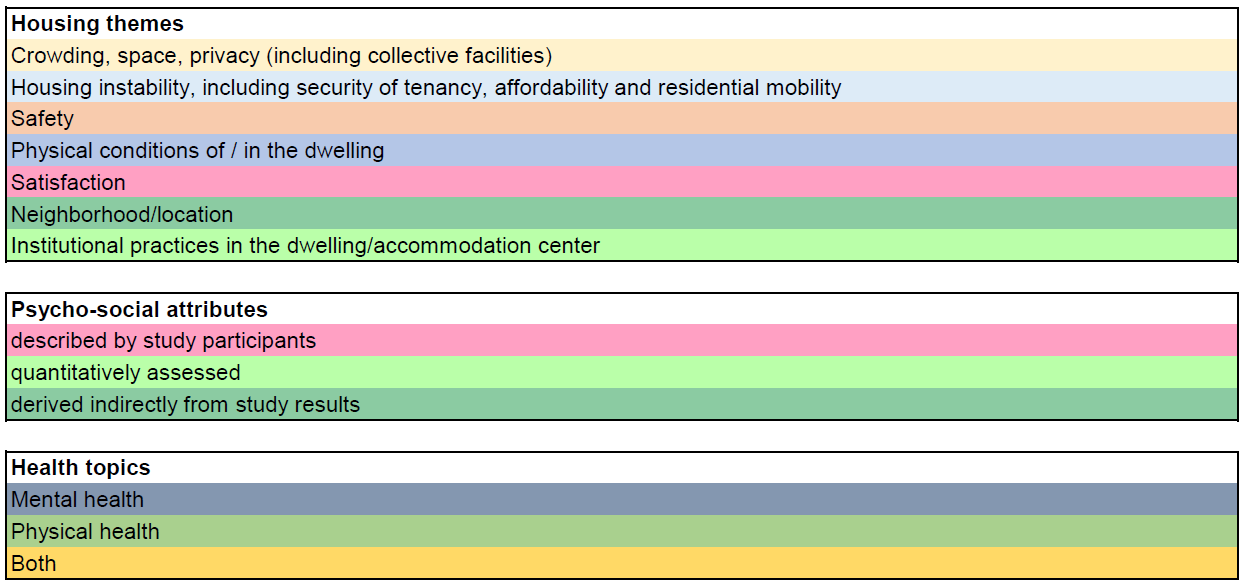
**

**Supplementary file 5: Conceptual map (Bielefeld, Germany, 2022)**

**
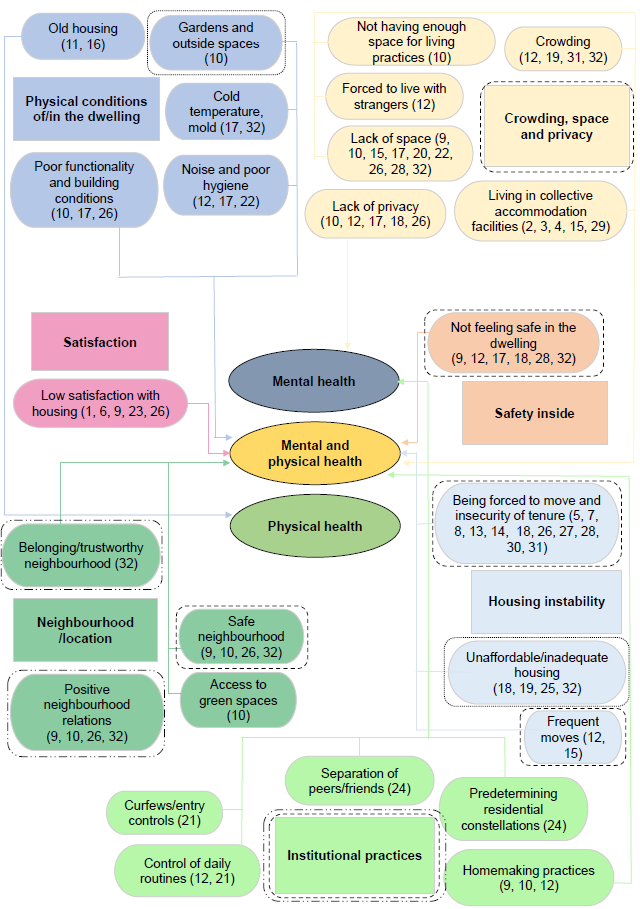
**

Psychosocial attributes

Demand

Control

Expressing status

**Supplementary file 6: Characteristics of studies included (Bielefeld, Germany, 2022)**


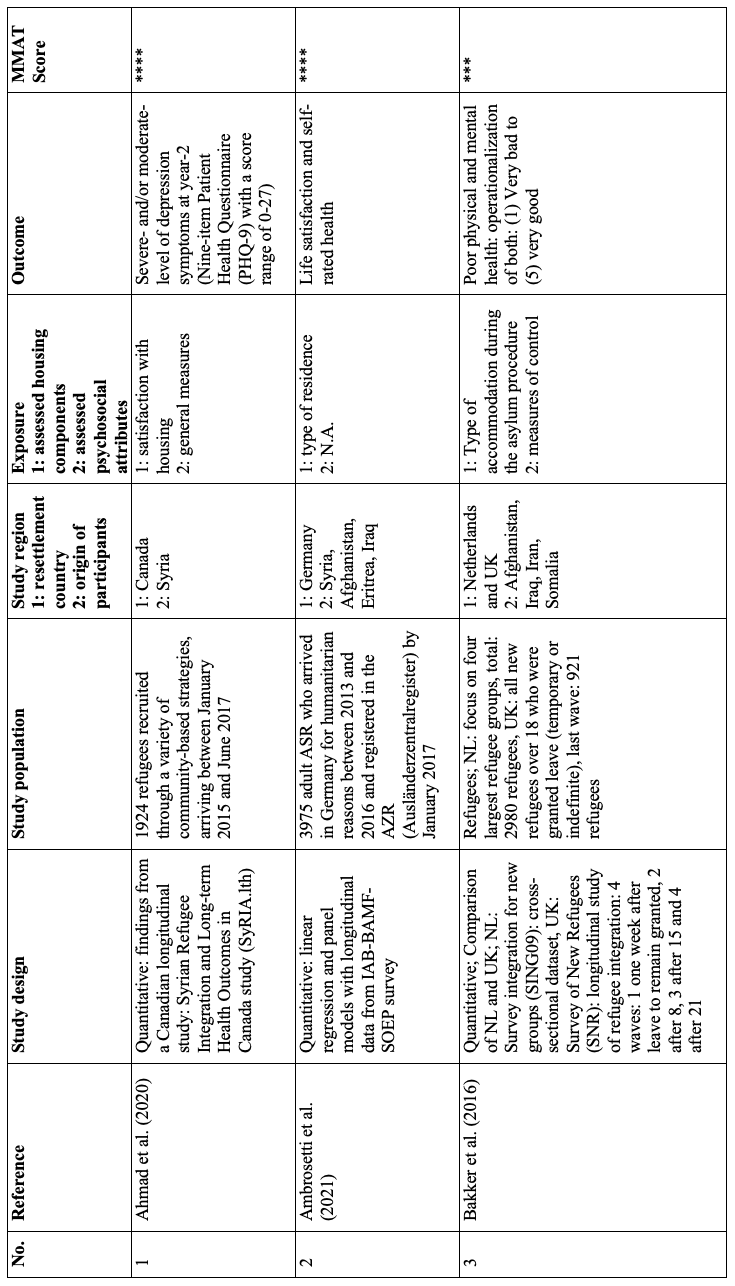

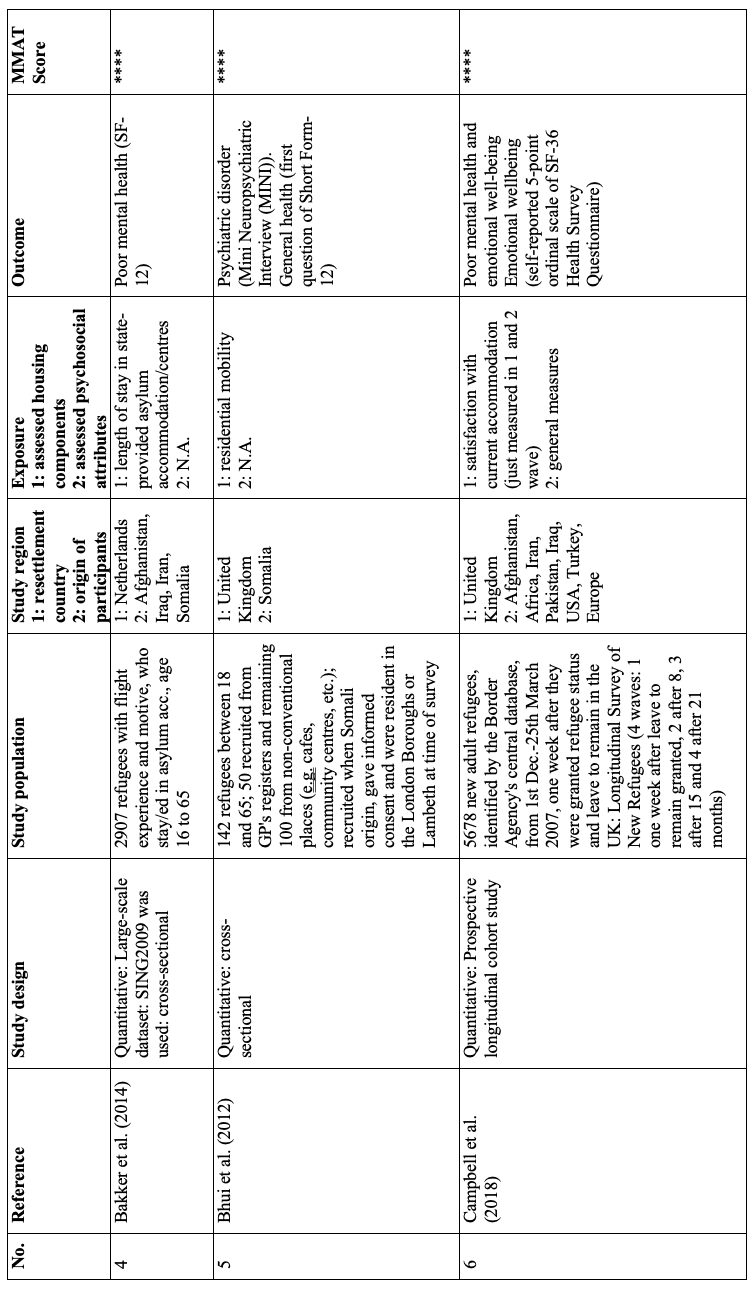

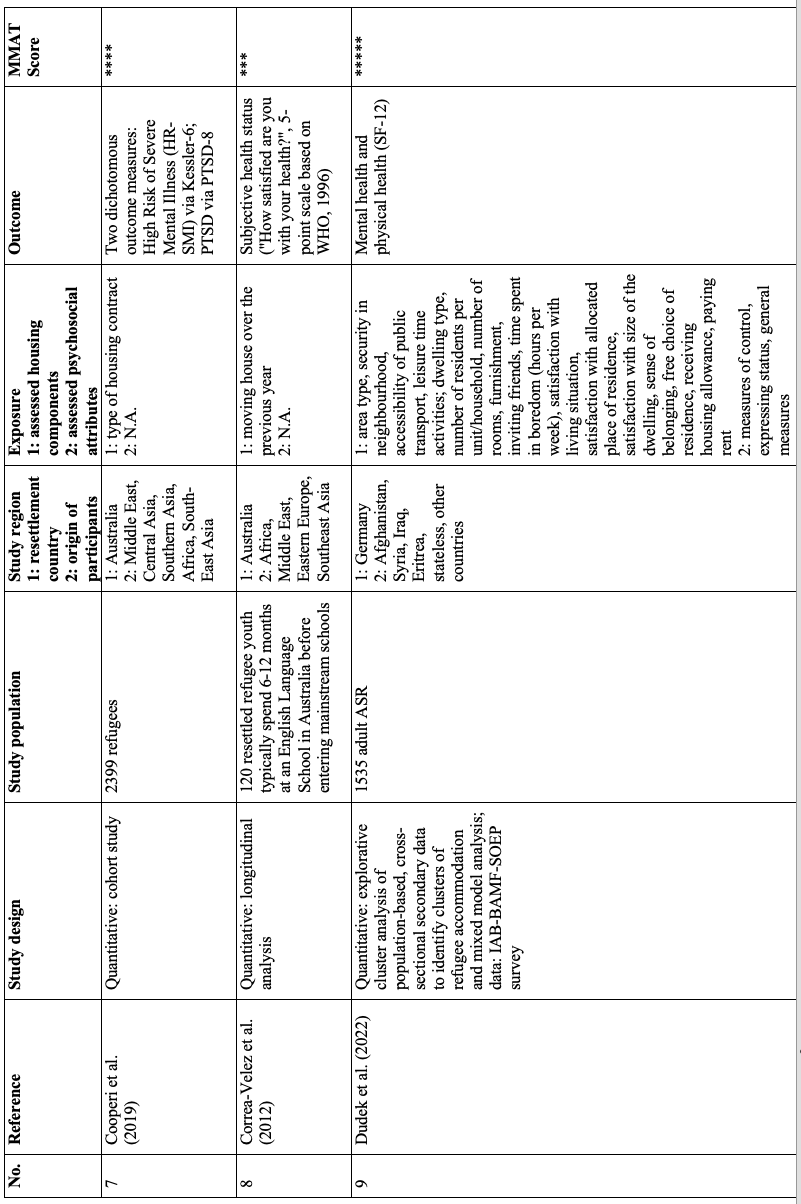

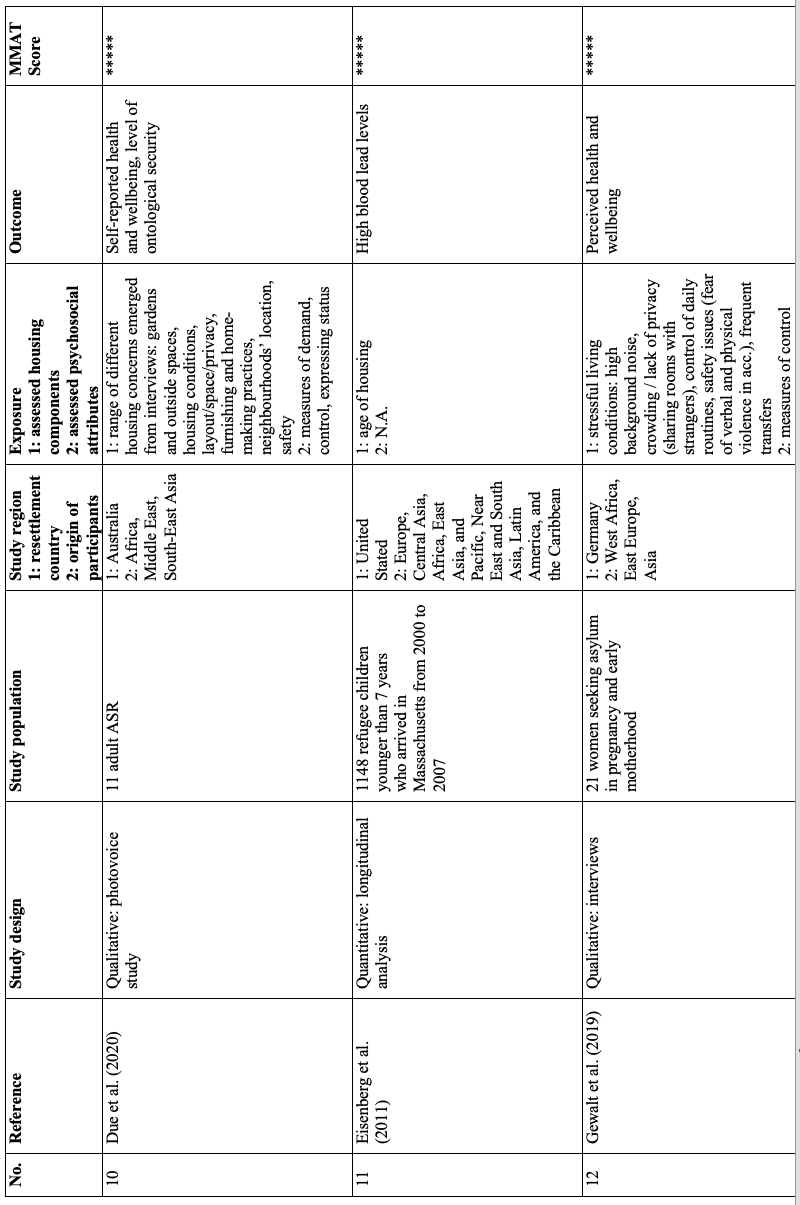

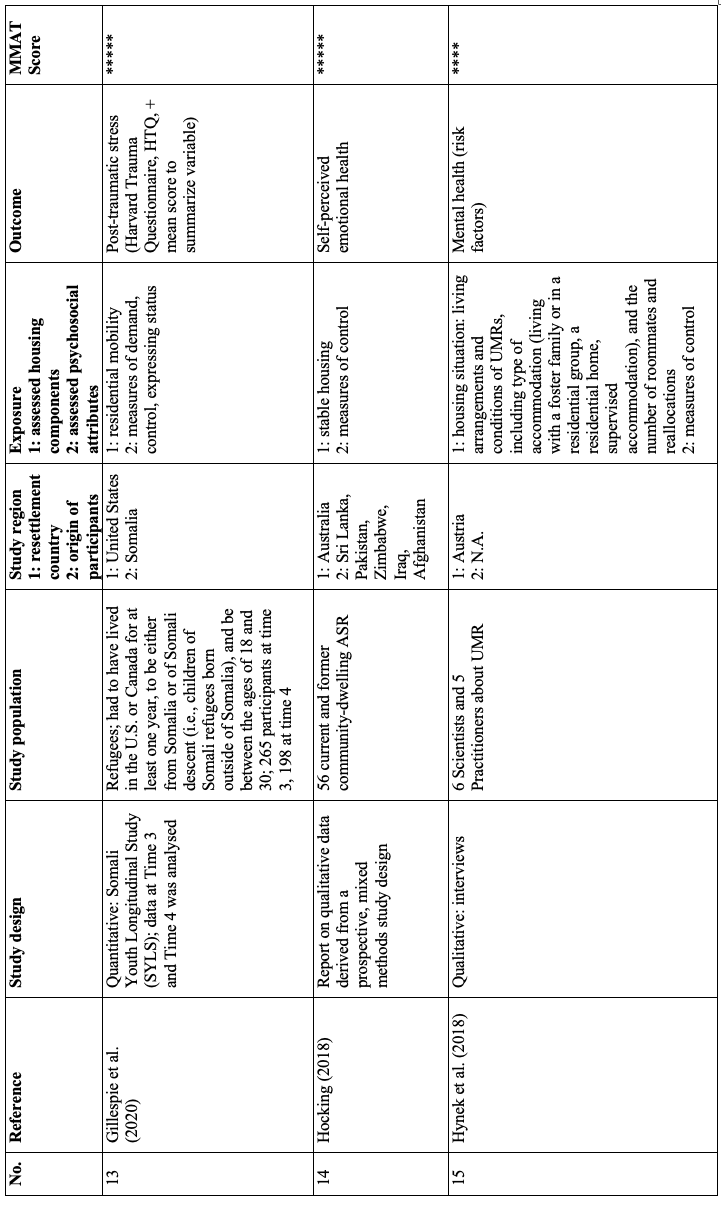

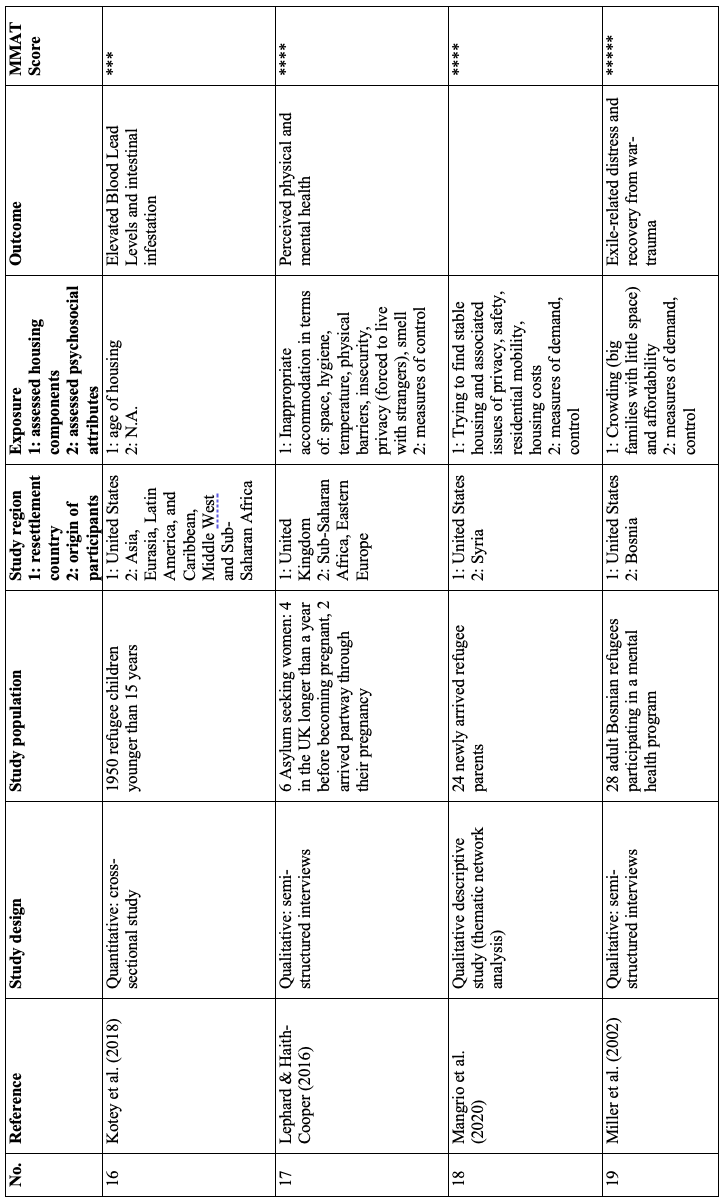

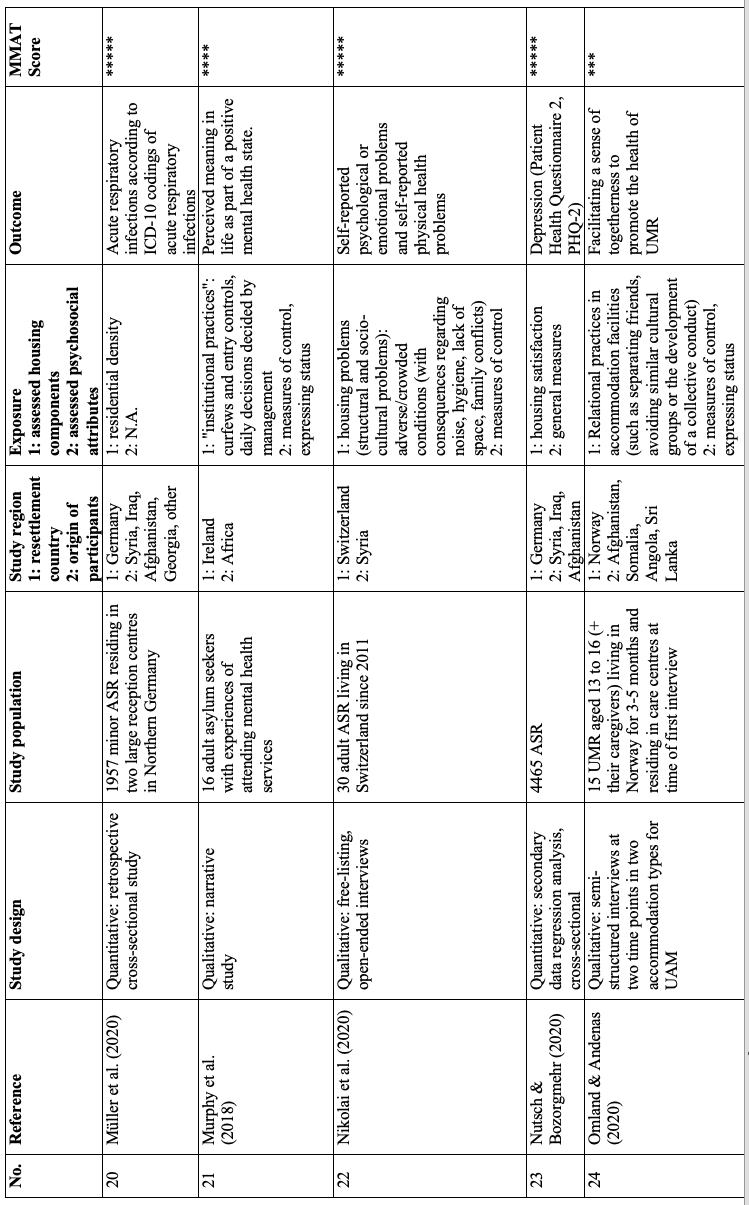

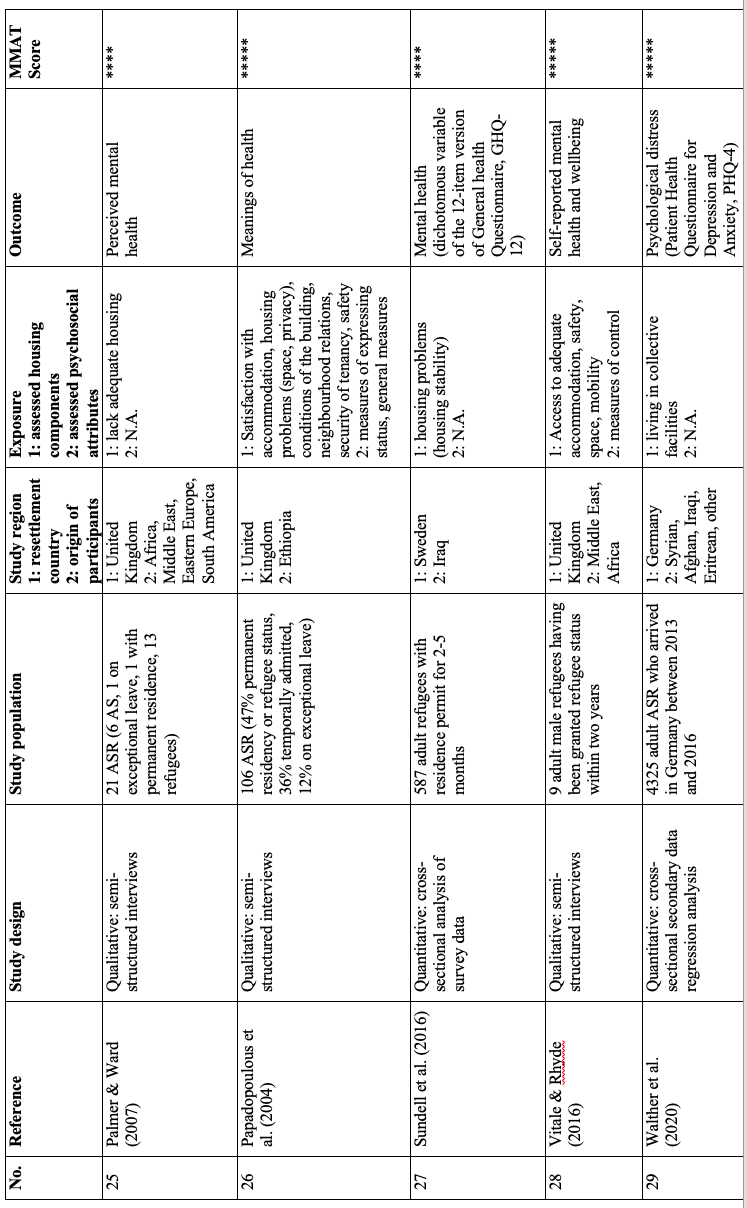

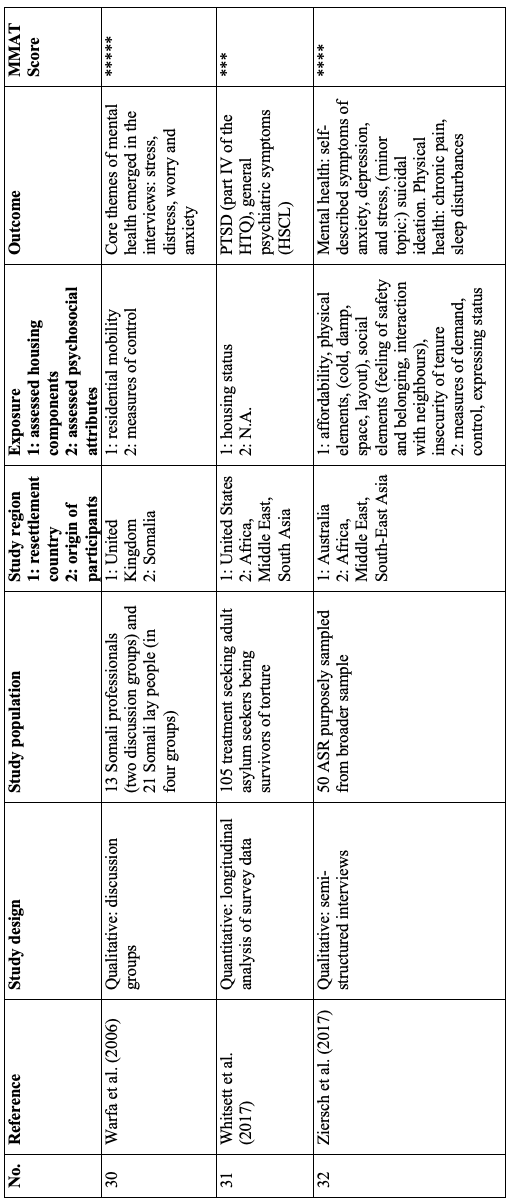


**Supplementary file 7: Quality assessment (Bielefeld, Germany, 2022)**

| **Screening criteria (for all types)** |
| --- |
| 1. Clear research question? (or: research aim is clearly specified) 🡪 yes / no / can’t tell |
| 1. Do collected data allow to address the research questions? 🡪 yes / no / can’t tell |
| 1. **Qualitative studies (5P.)** |
| - 1. Link to research question (1P.) |
| 1.2 Data collection methods (1P.) |
| 1.3 Findings and data (1P.) |
| 1.4 Interpretation of results? (1P.) |
| 1.5 Coherence (1P.) |
| 1. **Quantitative randomized controlled trials (5P.)** |
| 2.1 Randomization (1P.) |
| 2.2 Comparability of groups (1P.) |
| 2.3 Complete outcome data? (1P.) |
| 2.4 Outcome accessors (1P.) |
| 2.5 Adherement to intervention (1P.) |
| 1. **Quantitative non-randomized studies (5P.)** |
| 3.1 Representativeness (1P.) |
| 3.2 Appropriate measures (1P.) |
| 3.3 Complete outcome data (1P.) |
| 3.4 Confounders (1P.) |
| 3.5 Exposure (1P.) |
| 1. **Quantitative descriptive studies (5P.)** |
| 4.1 Sampling strategy (1P.) |
| 4.2 Representativeness (1P.) |
| 4.3 Appropriate measurements (1P.) |
| 4.4 Non-response bias (1P.) |
| 4.5 Statistical analysis (1P.) |
| 1. **Mixed methods studies (5P.)** |
| 5.1 Rationale for study design (1P.) |
| 5.2 Integration of data (1P.) |
| 5.3 Meta inference (1P.) |
| 5.4 Divergences and inconsistencies (1P.) |
| 5.5 Separate criteria (1P.) |


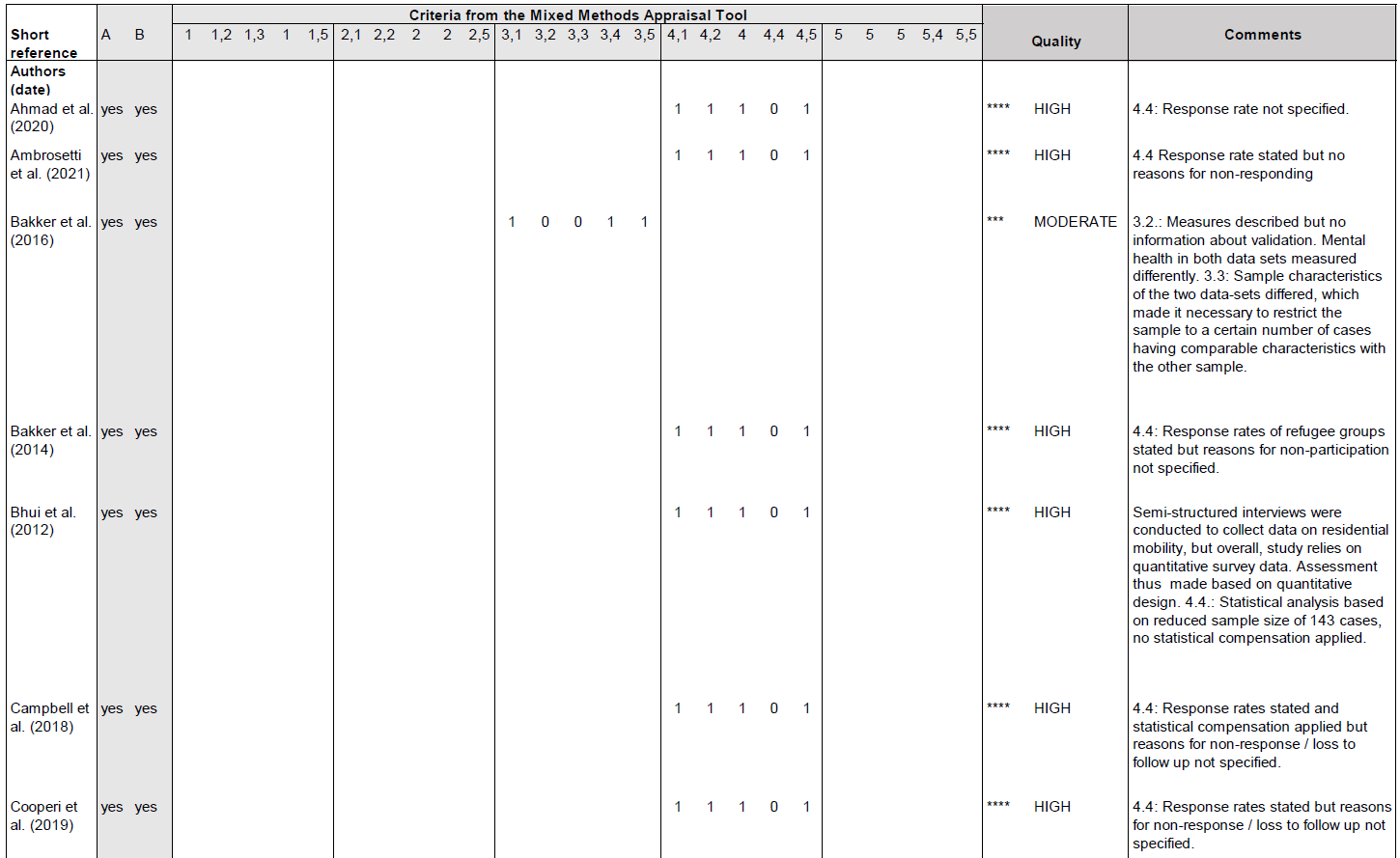


**
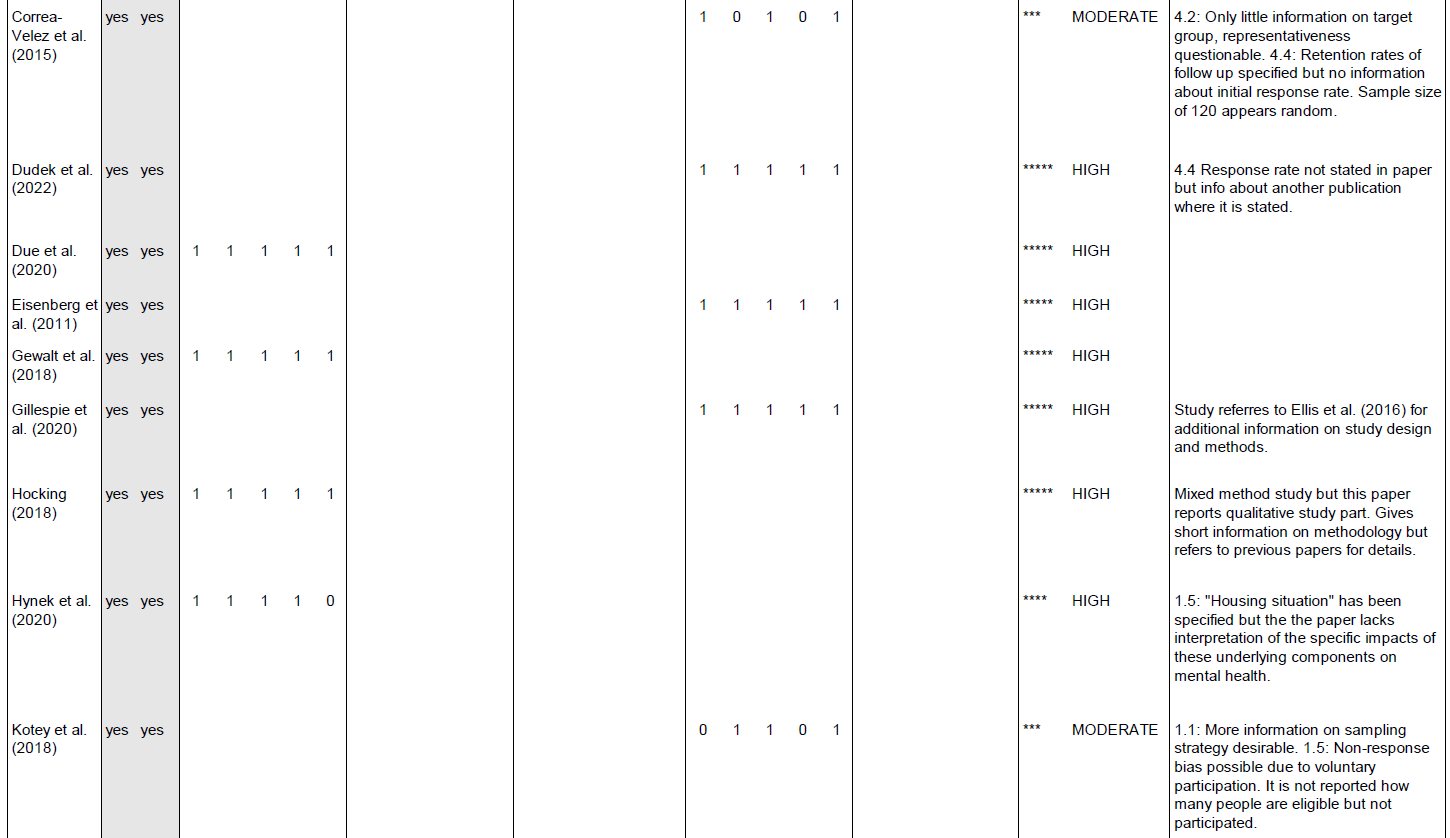
**

**
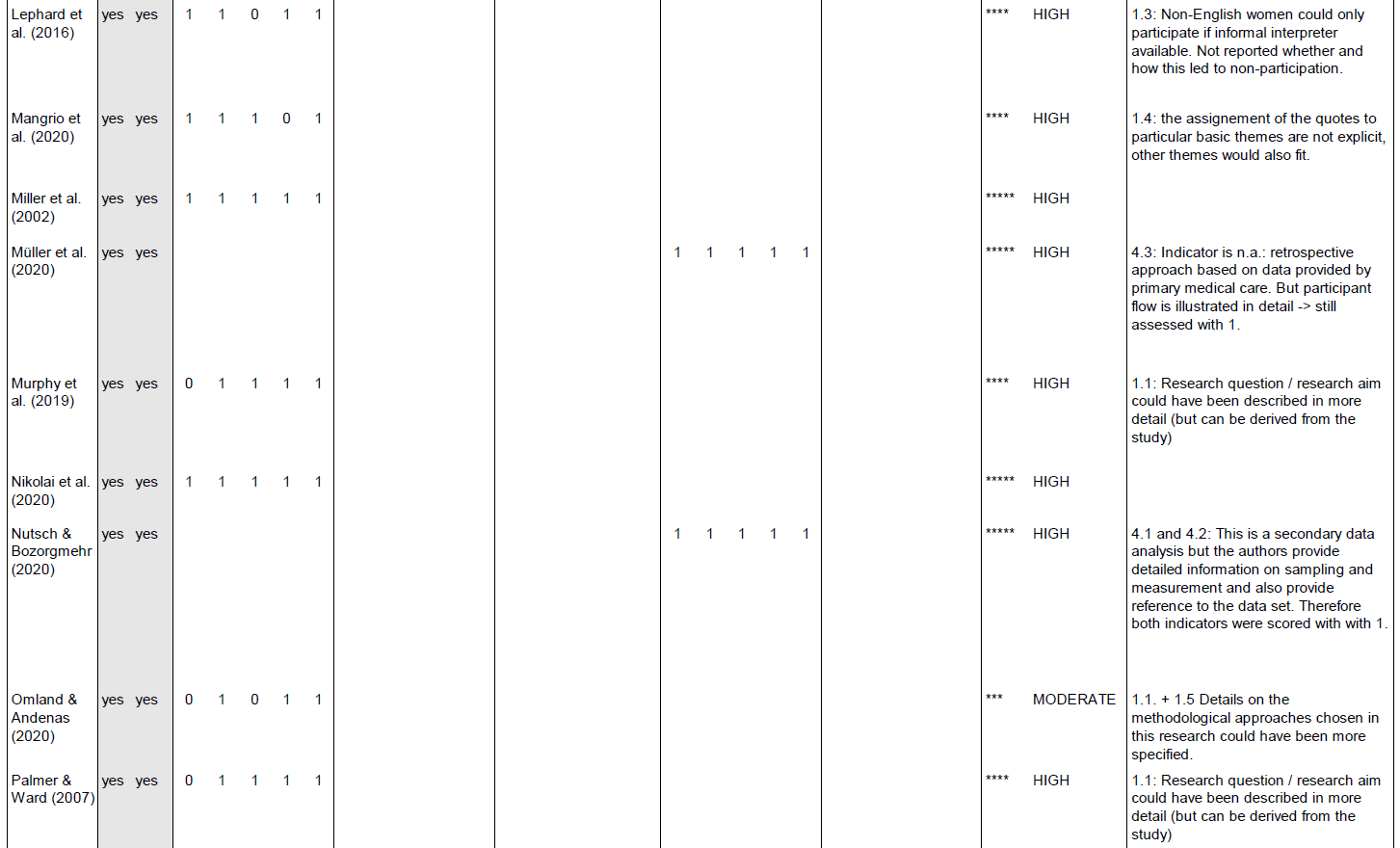
**

**
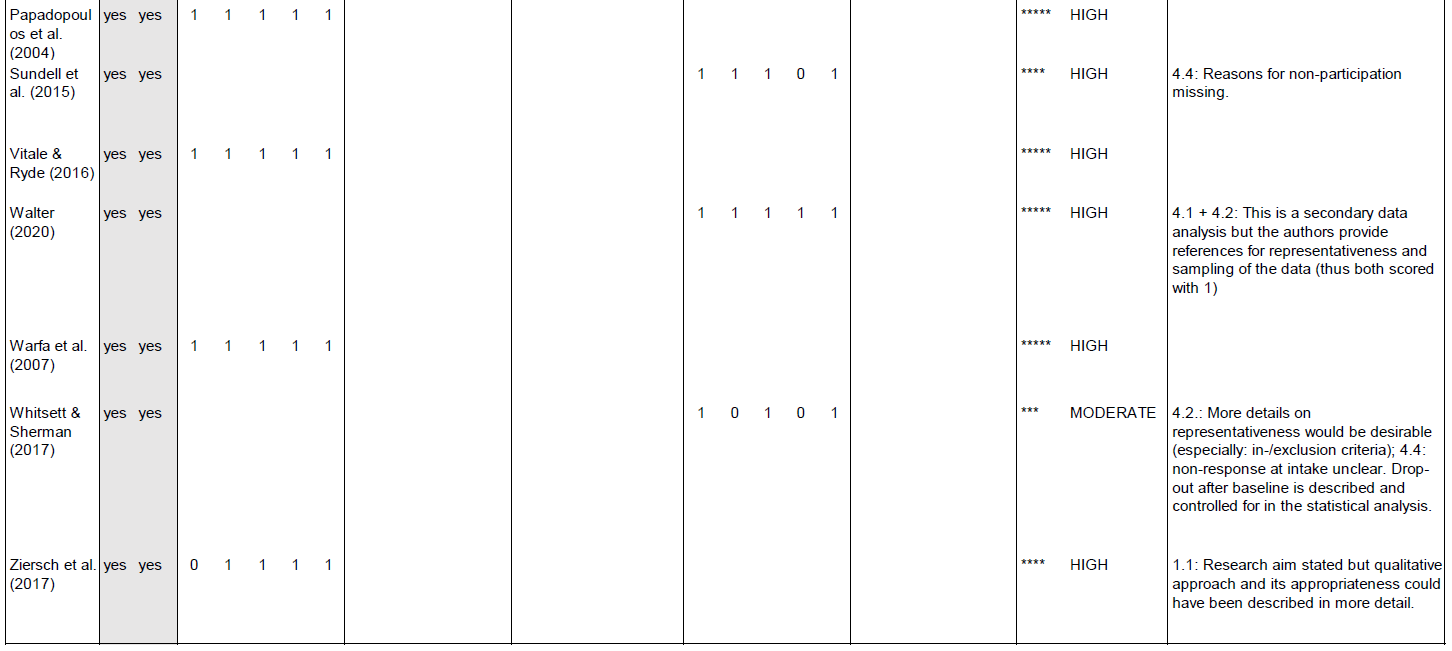
**
